# The effects of additives as a marinade producer on nutritional quality parameters of *Oncorhynchus mykiss* fish during storage at 4°C

**DOI:** 10.1002/fsn3.3745

**Published:** 2023-10-10

**Authors:** Ali Aberoumand, Abolfazl Aminimehr

**Affiliations:** ^1^ Department of Fisheries Behbahan Khatam Alanbia University of Technology Behbahan Iran; ^2^ Behbahan Khatam Alanbia University of Technology Behbahan Iran

**Keywords:** boiled and cold marinade, macronutrients, pH, rainbow trout, yield

## Abstract

The purpose of the present study was to evaluate the utilization of additive ingredients on the proximate composition, energy value, yield percentage, and pH value of cold and cooked Rainbow trout marinade. In this study, the effect of the marinating solution with different concentrations of lemon juice and salt on various quality properties in the processed muscle was determined. Results showed the pH value of the cold marinade in treatments 2, 3, and 4 decreased to 5.57, 5.15, and 5.14 with significant differences, respectively. The results showed that the marinade yield range (27.1%–19.28%) was found as a result of the marinating process. There was a significant difference between the cold and cooked fish marinades in terms of water content, pH, proximate contents, and yield percentage. The protein content in cold marinade was highest at 16.43%, while in treatments 2, 3, and 4, it decreased to 16.42%, 14.00%, and 14.56%, respectively with a significant difference. The lipid content in cold marinade (2.31%) for treatment 4 was highest, while in treatments 3 and 4, it increased significantly. The cold marinade treatment 4 found the highest energy and nutrient values, while the yield of marinade was 61.21%. The treatment T2 in cooked marinade found the highest energy value and the lowest weight loss (89.7%). Moisture retention in fish‐cooked marinade treatments was found to be 13.5%, 5.52%, and 9.77%. It can be concluded that treatment 2 was the best option for both cold and cooked marinades.

## Graphical abstract

Results showed the pH value of cold marinade in treatments 2, 3, and 4 decreased with a significant difference to 5.57, 5.15, and 5.14, respectively. The results showed that the marinate yield range (27.1%–19.28%) was found as a result of the marinating process. There were significant differences between the cold and cooked fish marinades in terms of water content, pH, proximate contents, and yield % in the treatments. The protein contents in cold marinade with 16.43% were highest, while in treatments 2, 3, and 4, they decreased to 16.42%, 14.00%, and 14.56%, respectively, with significant differences. The lipid contents in cold marinade (2.31%) for treatment 4 were highest, while in treatments 3 and 4, they increased with significant differences. The cold marinade treatment 4 found the highest energy and nutrient values, while the yield of marinade was 61.21%. The treatment T2 in cooked marinade found the highest energy value and the lowest weight loss (89.7%). Moisture retention of fish‐cooked marinade samples was determined at 13.5%, 5.52%, and 9.77%. It can be concluded that treatment 2 was the best option for both cold and cooked marinades.

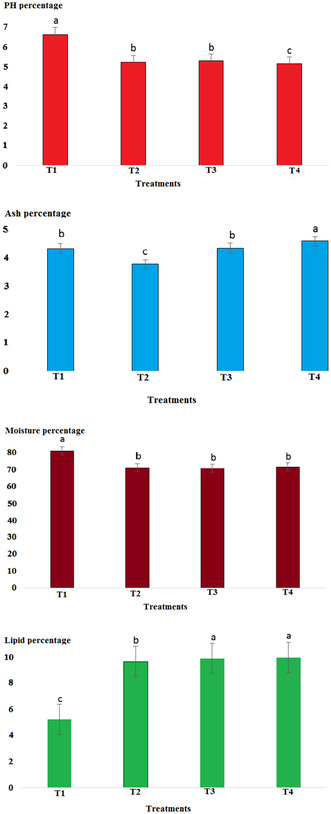

## 1 INTRODUCTION

Rainbow trout (*Oncorhynchus mykiss*) is one of the most important fishes consumed in southern Iran, in Behbahan city. Fatty fish such as Rainbow trout have a limited shelf life, and the quality deterioration of this species is mainly due to the rapid growth of microorganisms and lipid oxidation (Maktabi et al., 2016).

The growth of fisheries production has increased quickly in the last few years, and at the same time, there is an increase in consumers' need or demand for convenience fisheries products. “Ready‐to‐eat,” “ready‐to‐cook,” or “ready‐to‐serve” seafood in attractively packed convenience foods to match the dynamic desires of today's consumers running short of time are among such products. Marinating is a processed treatment with edible acids and salt that is put into brine, sauce, or oil. It involves an increase in ionic strength and a decrease in pH, resulting in a desirable change in the taste, texture, and flavor of marinades. Because most marinades contain acidic ingredients, the marinating should be done in a glass, ceramic, or stainless steel container. The food should be covered and refrigerated throughout the process (Sushri et al., 2020).

However, increased fish production leads to increased fish disposal costs. Therefore, keeping fish to use this food source can become more important than ever. For this purpose, fish fermentation can be a solution to increase the useful life of fresh fish (Halpern et al., 2019; Österblom et al., 2017).

In addition, the health benefits associated with the consumption of fermented fish may expand the market for fermented fish products. Future directions for fermented fish products include improving the fermentation process through modification and improvement of fermentation parameters. The breakdown of lipids and proteins is responsible for the positive and negative properties of fermented fish. To maximize the fermentation potential of fish, temperature, pH, and fermentation time should be optimized according to the species of fish used. In addition, more detailed studies should be conducted on the direct composition of fermented fish to provide a firmer foundation and guidance for improving its sensory properties (Kusi, 2023).

Fish marinades, which are popular in many parts of the world, are prepared by cold‐ripening fish meat in an aqueous solution of table salt (4%–9%) and acetic acid (3%–7%) called brine. The ripening of marinated fish meat takes place as a result of numerous physical, biochemical, and microbiological changes. Most of these transformations are related to the hydrolysis of proteins and lipids and their interactions. In low‐pH marinades, proteases such as cathepsin D and E and pepsin, as well as cathepsin B and L, are active (Szymczak et al., 2018). In the first 100 h of the marinating process, the fastest increase in the content of peptides is observed, which is due to the activity of endopeptidases, while after 5 to 7 days, the increase in the content of free amino acids may increase as a result of the activity of exopeptidases (Szymczak et al., 2018). Protein hydrolysis products, especially those with low molecular weight, are responsible for the sensory characteristics of marinated meat. Significant loosening of meat structure is also caused by the dissolution of weakly cross‐linked collagen in acetic acid (Szymczak et al., 2018).

Marinating is a fish meat processing method that involves immersion in cooked or uncooked liquid marinade compositions, which may contain various additives such as acids, enzymes, and spices (Lopes et al., 2022). It is also a technique to soften fish meat using biological, chemical, and physical methods (Gomez et al., 2020). Marinades are mixtures of components used to improve the color, flavor, texture, palatability, and tenderness of fish and seafood (Gomez et al., 2019). Over time, different types of ingredients have been used in marinades, changing the main purpose from preserving or marinating to softening and flavor enhancement (Lopes et al., 2022). Fish is treated with different types of biological marinades (Latoch et al., 2019), turmeric, burnt ginger, karuk juice (Ozturk & Sengun, 2019), red chili, pepper, garlic, and ginger (Gamage et al., 2017). Various chemicals are used in pickling to enhance fish muscle properties, including citric acid, acetic acid, tartaric acid, apple cider vinegar, and sodium chloride (NaCl) (Gomez et al., 2020; Gómez‐Salazar et al., 2018). Ingredients used in marinade formulation are critical to achieving the desired sensory properties, such as flavor and texture, which ultimately determine the quality of the final product. They improve the natural rate of proteolysis in fish meat by drastically reducing the pH, thereby stimulating enzymatic and proteolytic activities in the muscle. The use of different acids helps to break down and soften the meat, while seasoning and spices add flavor to the meat. In addition to contributing to sensory properties, they have an effective effect on enzymes and the inactivation or inhibition of pathogenic and spoilage microorganisms to increase the shelf life of fish meat. These effects can be associated with compounds such as polyphenols, organic acids, ethanol, and antimicrobial agents (Latoch, 2020; Lopes et al., 2022).

The quality and acceptability of marinated products are mainly, if not solely, dependent on how the maturation process proceeds. In addition, the raw material used also determines the quality of the final product and the technological process of marinating, given that it is a process without heat treatment and is dependent on the degree of ripening (Simora et al., 2021).

The ripening of the marinade fillet is the result of numerous physical, biochemical, and microbiological changes. Most of these transformations are related to the hydrolysis of proteins and lipids and their interactions. In low‐pH marinades, aspartic acid proteases such as cathepsin D and E and pepsin are active, as are cysteine proteases such as cathepsin B and L. Protein hydrolysis products, especially those with low molecular weight, are responsible for the sensory properties of pickled meat. Significant loosening of the meat structure is also due to the dissolution of collagen with weak cross‐linking in acetic acid (Szymczak et al., 2018). The comparison between the nutrient compounds of fresh, cooked, and cold marinated *O. mykiss* and the effects of processing on them has not yet been explored scientifically. The main objective of the present study was to estimate the effect of the marinating method on the nutrients, compounds, and pH of marinated *O. mykiss*.

## 2 MATERIALS AND METHODS

### 2.1 Experiment design

Formulation and proximate composition of the experimental diets are shown in Table 1.

**TABLE 1:** Formulation and proximate composition of the experimental diets.

Ingredient	Content (air‐dry matter, %)
Fish meal	4
Soybean meal	30
Wheat middling	30
Rapeseed meal	22
Soybean oil	2.5
CMC (carboxymethyl cellulose)	2.2
Vitamin premix	3
Trace mineral premix	3
Dicalcium phosphate	2
Choline chloride	0.6
Lysine	0.7
Total	100
Proximate composition	
Crude protein	28.14
Crude lipid	4.53
Total phosphorus	0.98
Lysine	1.63

This research was conducted in 2022 at Behbahan Khatam Alanbia University of Technology, Iran. The fresh fish, Ghezelala with the scientific name *O. mykiss*, was purchased from the Behbahan fish market in Iran, then placed in an ice box and transferred to the laboratory. The fresh fish was washed with distilled water, and its biometrics were measured. The total length of the fish was 42 cm, its body width was 10 cm, and its weight was 756.2 g. The fish wastes, such as the head, tail, intestines, and viscera, were removed and the fillet measured. The net weight of the fillet was 421.1 g, and the yield of the fish fillet was 55.69%.

Two cold and cooked marinating processes were used to prepare the marinade. To prepare the cold marinade, the raw fish fillets were placed in the marinade solution without heat treatment, and then the whole raw fillets were processed in the 300 mL marinade solution with treatments 1, 2, and 3 separately.

To prepare the cooked marinade, the fresh fillet was cooked in boiling distilled water for 20 min and then cooled. Then the cooked whole fillets were placed in the 300 mL marinade solution for treatments 1, 2, and 3 separately. The control treatment without immersion in the marinade solution was analyzed. The cooked and cold marinades were prepared separately (Simora et al., 2021).

### 2.2 Preparation of a marinade solution

First, for treatment 3, 25 g of lemon juice and 12.5 g of freshly grated garlic, 3 g of salt, 0.15 g of turmeric, 0.05 g of red pepper powder, 0.15 g of black pepper powder, and 5 mL of distilled water were mixed and homogenized, while for treatment 2, lemon juice 4% and salt 8%, and for treatment 3, lemon juice 5% and salt 10%, and for treatment 4, lemon juice 6% and salt 12% were used (Simora et al., 2021).

For two marinating processes, three treatments and one control were used. For cooked marinade processing, 45 g of fillet was used for each treatment, while for cold marinade, 50 g of fillet was used for each treatment. Then the fillet was placed in a plastic container containing the marinade solution, and the lid was closed while it was completely immersed. The containers containing the fillet and the marinade solution were kept at 4°C for 4 days. Sample analysis was performed on the 1st and 4th days of storage for all treatments and controls. Analysis of macronutrients, pH, and weight gain were carried out.

### 2.3 Composition analysis

Prior to storage the samples at refrigerated temperature, composite fresh samples of the marinated and un‐marinated fish fillets were analyzed for moisture, protein, ash, and lipid based on the AOAC (2005) methods. All samples were conducted in triplicate and all reagents were of analytical grade.

### 2.4 Determination of weight loss and increase

The weight loss and increase were considered during storage. The weight loss and increase in the marinade fillet were carried out according to the method of Larsen et al. (2008) as follows:

$$\begin{array}{c} \text{The weight loss or increase}\;\left(\%\right)\\ =100\times \left(g\;\text{fillet before processing}–g\;\text{fillet after processing}\right)/\left(g\;\text{fillet before processing}\right). \end{array}$$

### 2.5 Determination of moisture retention

Moisture retention%=%yield×%moisture in cooked marinade/100.

### 2.6 Determination of moisture content

Precisely 5 g of each of the samples were weighed and dried in the oven at 105°C for 3 h to a constant weight. The moisture content was measured using the below formula:

$$\text{Moisture content}=w_{2}-w_{3}/w_{2}-w_{1}\times 100,$$

where *w*_1_ = weight of empty dish, *w*_2_ = weight of dish and sample before drying, and *w*_3_ = weight of dish and sample after drying.

### 2.7 Determination of ash content

The ash content was determined by igniting 10 g of dry sample in a muffle furnace at 600°C. It was cooled in a desiccator and weighed. The ash content was reported according to the below formula.

$$\text{Percentage}\;\mathrm{ash}=w_{2}-w_{3}/w_{1}\times 100.$$

where *w*_1_ = weight of the sample, *w*_2_ = weight of sample + crucible, and *w*_3_ = weight of sample + crucible (constant weight after drying).

### 2.8 Determination of crude protein

A 10 g of sample was weighed and digested in the macro‐Kjeldahl apparatus with concentrated sulfuric acid. The ammonia liberated from the resulting ammonium sulphate after adding sodium hydroxide was distilled into 1 M boric acid and then titrated with 0.1 M HCl. The nitrogen value was multiplied by 6.25 (protein factor) to obtain the value of the crude protein (AOAC, 2005).

### 2.9 Determination of fat

The crude fat was extracted from 10 g of each sample using a solvent extraction apparatus (Soxhlet apparatus) with low boiling point petroleum ether. The importance of the lipid obtained after evaporating off the solvent from the extract gave the weight of the lipid present in the sample (AOAC, 2005).

### 2.10 pH measurement

Five gram from each sample was homogenized separately by a homogenizer and mixed with 45 mL of distilled water. The pH value of each sample was measured by a PHS_550 digital device (made in China) in laboratory.

### 2.11 Statistical analysis method

SPSS software was used to statistically analyze the data, and the normality of the data distribution was checked by the Kolmogorov–Smirnov method. Using a one‐way ANOVA test, the presence or absence of differences between treatments was examined, and after observing a significant difference, the Duncan test was at a 95% confidence level to check the significance of differences between treatments.

## 3 RESULTS AND DISCUSSION

### 3.1 Proximate composition

**FIGURE 1:**
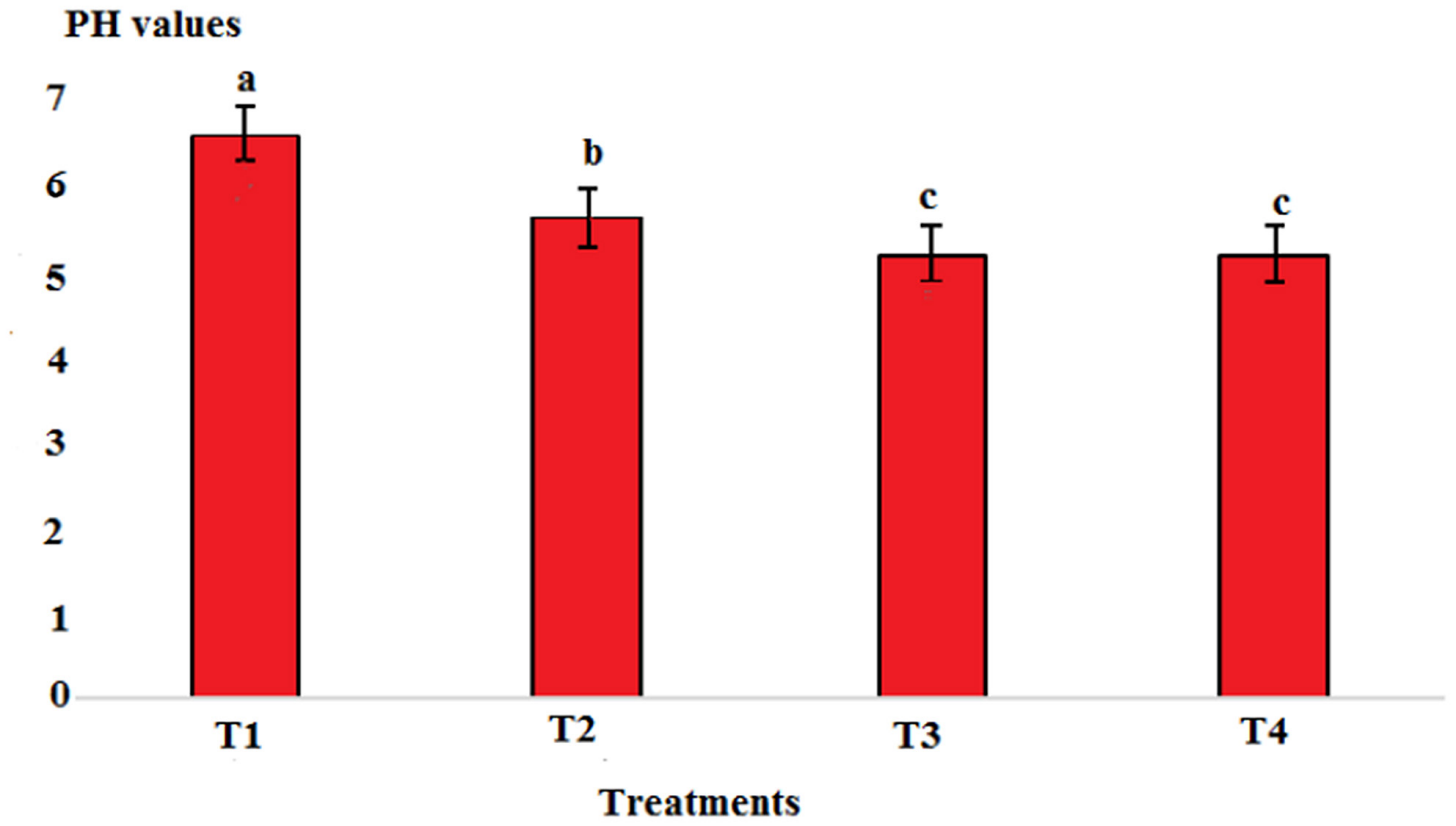
Comparison of pH of different cold marinade treatments with control. T1 was the control. For the three treatments, T2, T3, and T4, 25 g of lemon juice, 12.5 g of freshly grated garlic, 3 g of salt, 0.15 g of turmeric, 0.05 g of red pepper powder, 0.15 g of black pepper powder, and 5 cc of distilled water were mixed and homogenized. The ratio of lemon juice and salt was 1 to 2. For treatment 1, lemon juice 4% and salt 8%; for treatment 2, lemon juice 5% and salt 10%; and for treatment 3, lemon juice 6% and salt 12% were used.

**FIGURE 2:**
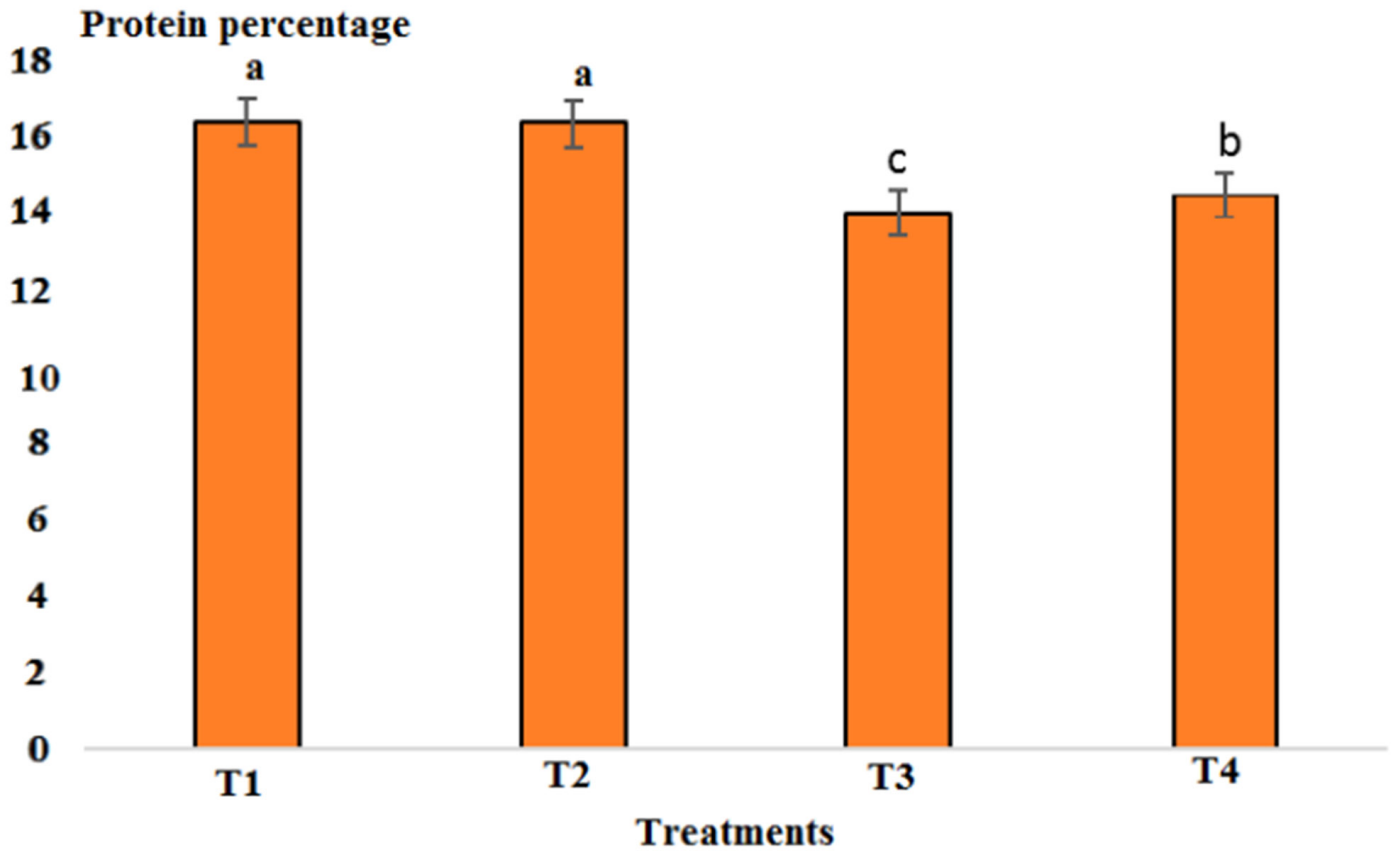
Comparison of protein contents of different cold marinade treatments with control. T1 was the control. For three treatments, T2, T3, and T4, 25 g of lemon juice and 12.5 g of freshly grated garlic, 3 g of salt, 0.15 g of turmeric, 0.05 g of red pepper powder, 0.15 g of black pepper powder, and 5 cc of distilled water were mixed and homogenized. The ratio of lemon juice and salt was 1 to 2. For treatment 1, lemon juice 4% and salt 8%; for treatment 2, lemon juice 5% and salt 10%; and for treatment 3, lemon juice 6% and salt 12% were used.

**FIGURE 3:**
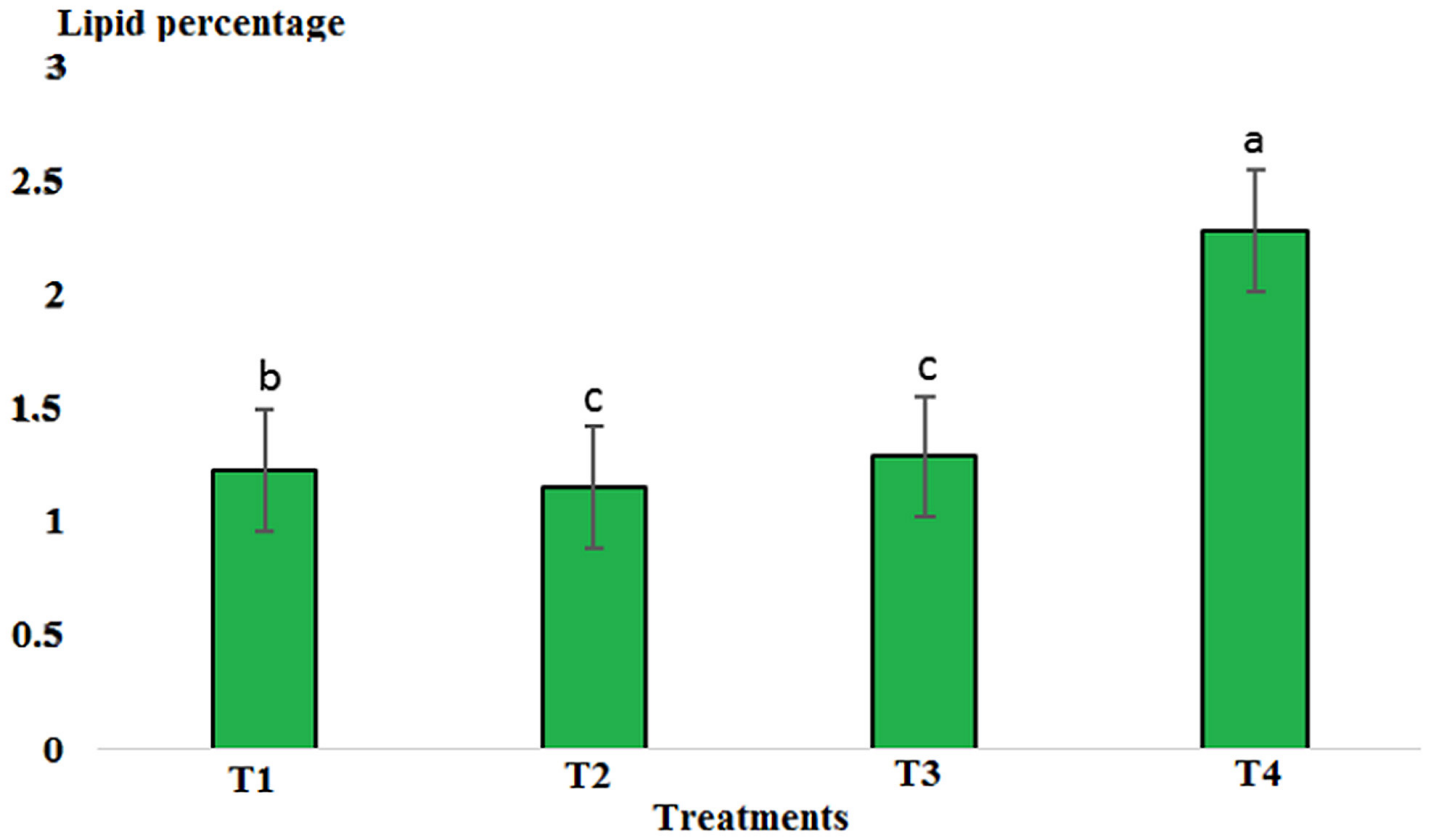
Comparison of lipid contents of different cold marinade treatments with control. T1 was the control. For the three treatments, T2, T3, and T4, 25 g of lemon juice, 12.5g of freshly grated garlic, 3 g of salt, 0.15 g of turmeric, 0.05 g of red pepper powder, 0.15 g of black pepper powder, and 5 cc of distilled water were mixed and homogenized. The ratio of lemon juice and salt was 1 to 2. For treatment 1, lemon juice 4% and salt 8%; for treatment 2, lemon juice 5% and salt 10%; and for treatment 3, lemon juice 6% and salt 12% were used.

**FIGURE 4:**
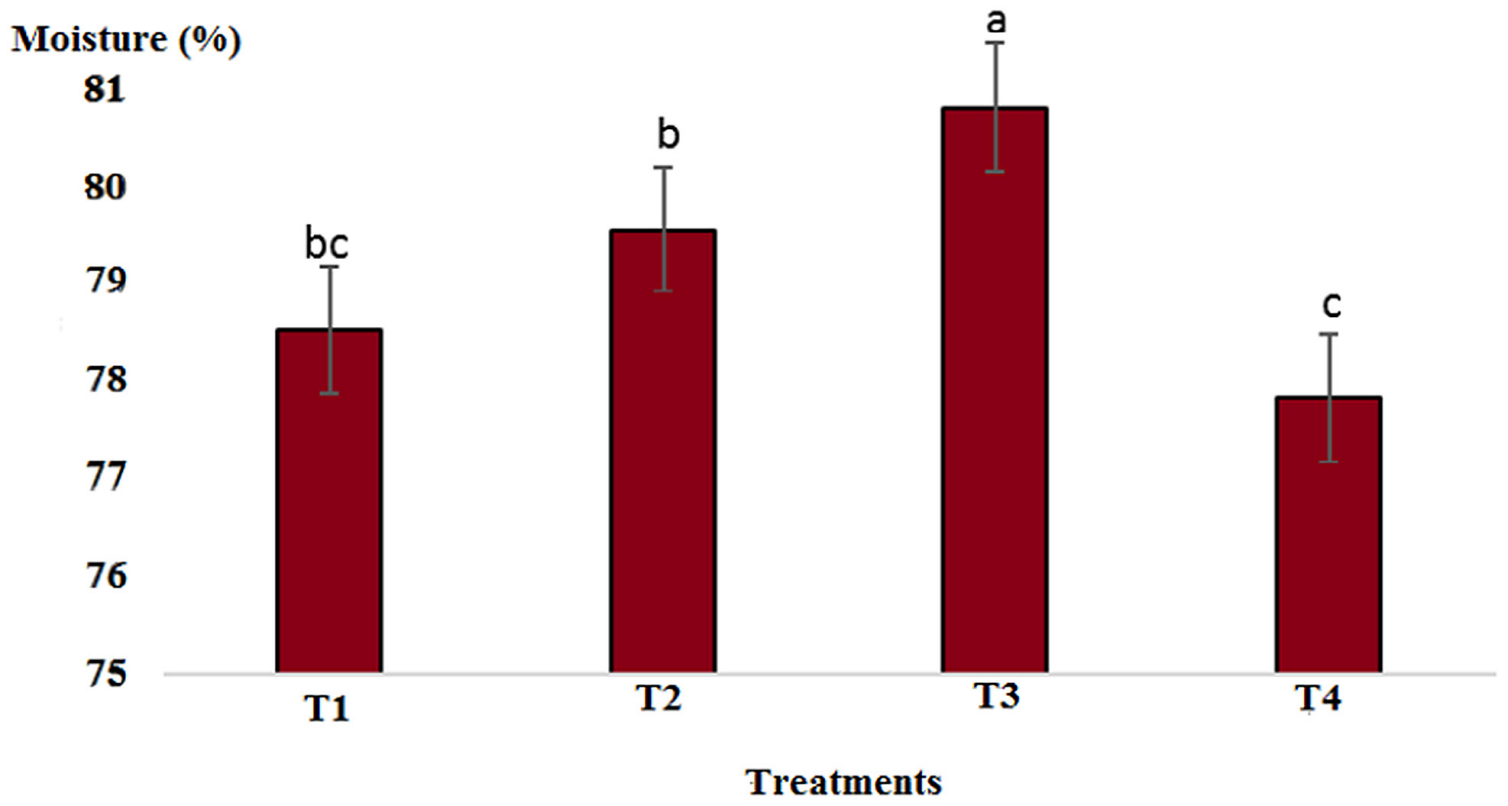
Comparison of moisture contents of different cold marinade treatments with control. T1 was the control. For the three treatments, T2, T3, and T4, 25 g of lemon juice, 12.5 g of freshly grated garlic, 3 g of salt, 0.15 g of turmeric, 0.05 g of red pepper powder, 0.15 g of black pepper powder, and 5 cc of distilled water were mixed and homogenized. The ratio of lemon juice and salt was 1 to 2. For treatment 1, lemon juice 4% and salt 8%; for treatment 2, lemon juice 5% and salt 10%; and for treatment 3, lemon juice 6% and salt 12% were used.

**FIGURE 5:**
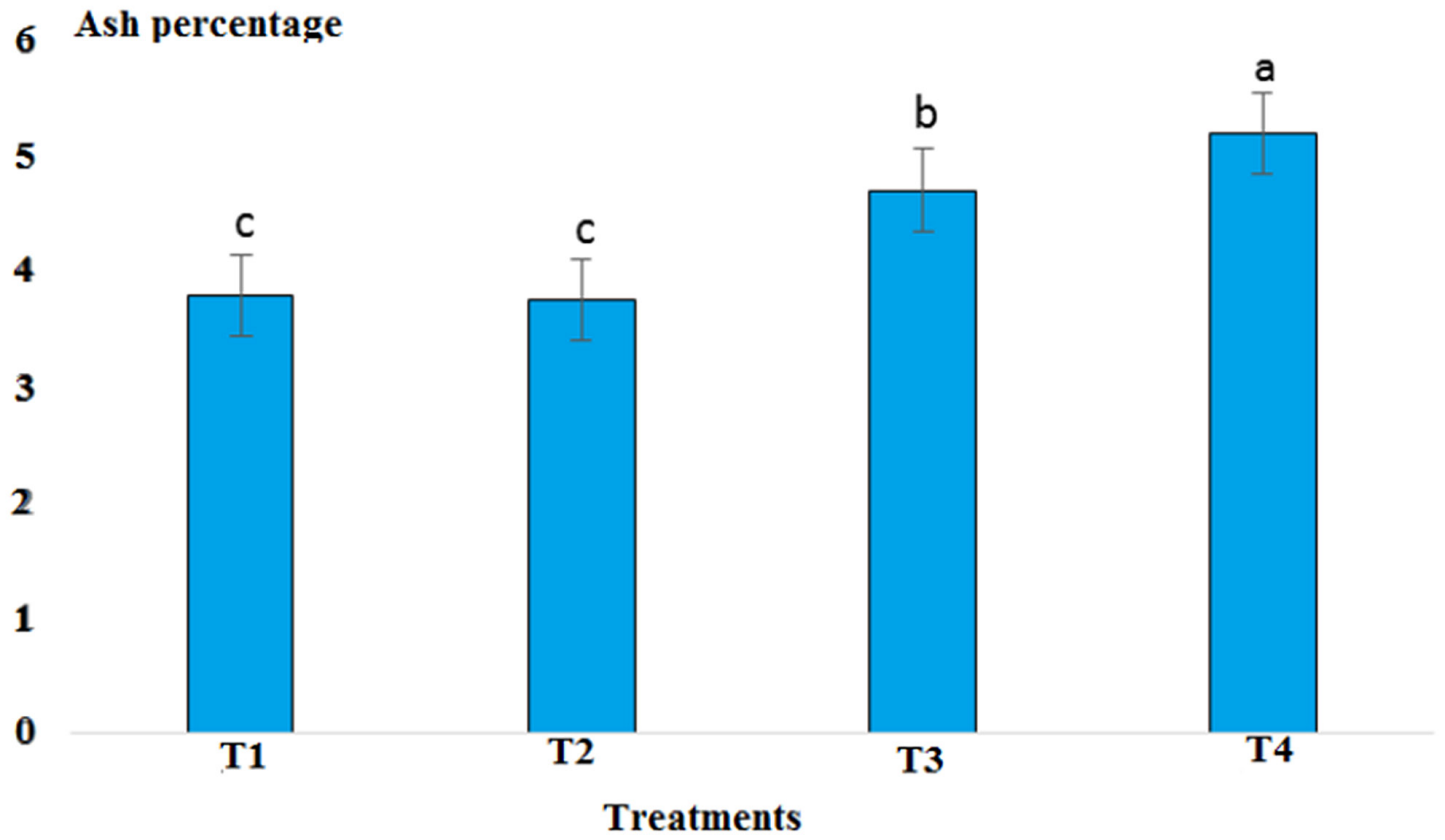
Comparison of ash contents of different cold marinade treatments with control. T1 was the control. For three treatments, T2, T3, and T4, 25 g of lemon juice, 12.5 g of freshly grated garlic, 3 g of salt, 0.15 g of turmeric, 0.05 g of red pepper powder, 0.15 g of black pepper powder, and 5 cc of distilled water were mixed and homogenized. The ratio of lemon juice and salt was 1 to 2. For treatment 1, lemon juice 4% and salt 8%; for treatment 2, lemon juice 5% and salt 10%; and for treatment 3, lemon juice 6% and salt 12% were used.

**FIGURE 6:**
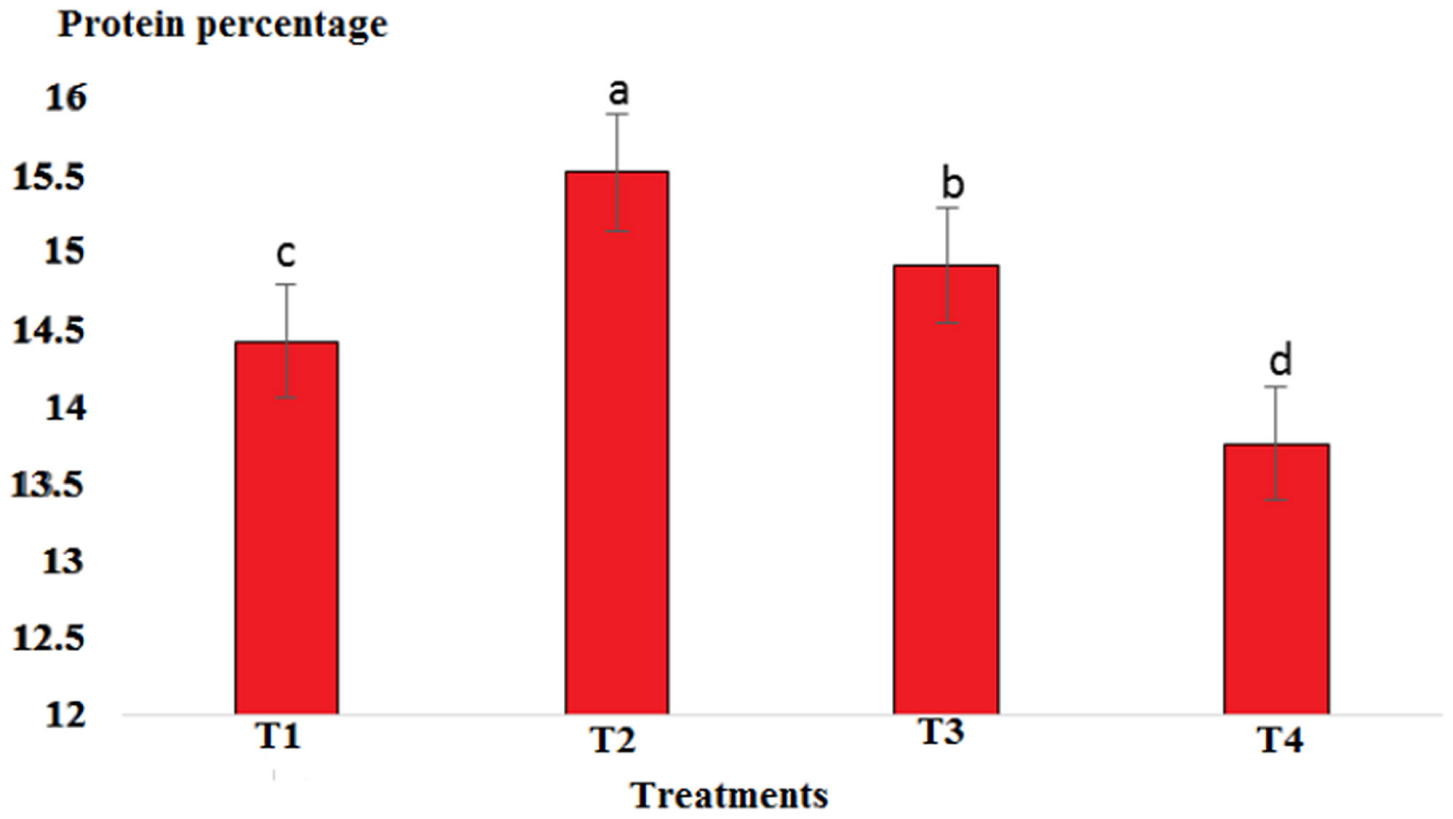
Comparison of protein contents of different cooked marinade treatments with control. T1 was the control. For three treatments, T2, T3, and T4, 25 g of lemon juice, 12.5 g of freshly grated garlic, 3 g of salt, 0.15 g of turmeric, 0.05 g of red pepper powder, 0.15 g of black pepper powder, and 5 cc of distilled water were mixed and homogenized. The ratio of lemon juice and salt was 1 to 2. For treatment 1, lemon juice 4% and salt 8%; for treatment 2, lemon juice 5% and salt 10%; and for treatment 3, lemon juice 6% and salt 12% were used.

Results showed that pH in cold marinade samples for control (6.25) was highest, while in treatments 2, 3, and 4, it decreased to 5.57, 5.15, and 5.14, respectively (*p* < .05). There was no significant difference between T3 and T4, but there was a significant difference between T2 and other treatments (*p* < .05). The decrease in pH value was due to the effect of acidic compounds in the marinade solution (Figure 1). The protein content in the cold marinade for the control (16.43%) was highest, while in T2, T3, and T4, it decreased to 16.42%, 14.00%, and 14.56%, respectively, with a significant difference between T3 and T4 compared to the control (*p* < .05). A slight decrease in protein content can be due to the breakdown of protein under the influence of the marinade solution compounds (Figure 2). The lipid content in cold marinade in T4 with 2.31% was highest, while in T3 and T4 it increased. There was a significant difference between T4 and the other treatments (*p* < .05). An increase in the fat content of the marinade can be the result of a decrease in moisture content during the marinade process (Figure 3). The moisture content of the cold marinade in T3 with 81% was highest, while it decreased in T4. There was a significant difference between T2, T3, and T4 (*p* < .05). The decrease in the moisture content can be the result of the effect of moisture‐absorbing compounds in the marinade solution on the fillet and the removal of water (Figure 4). The ash content in cold marinade in T4 with 5.2% was highest, while in T3 and T2, it was 4.72% and 3.77%, respectively. There was a significant difference between T3 and T4 compared to the treatments (*p* < .05). Increasing the ash content can result in the absorption of material in the marinade solution in the fillet (Figure 5). The protein content in cooked marinade for T2 at 15.54% was highest, while in T3 and T4, it was 14.92% and 13.76%, respectively, with a gradual decrease trend. There was a significant difference between T2, T3, and T4 compared to the control (*p* < .05). A sharp decrease in the protein content in the T4‐cooked marinade can be the result of the effect of the cooking on the denaturation of fillet myofibrillar protein (Figure 6).

**FIGURE 7:**
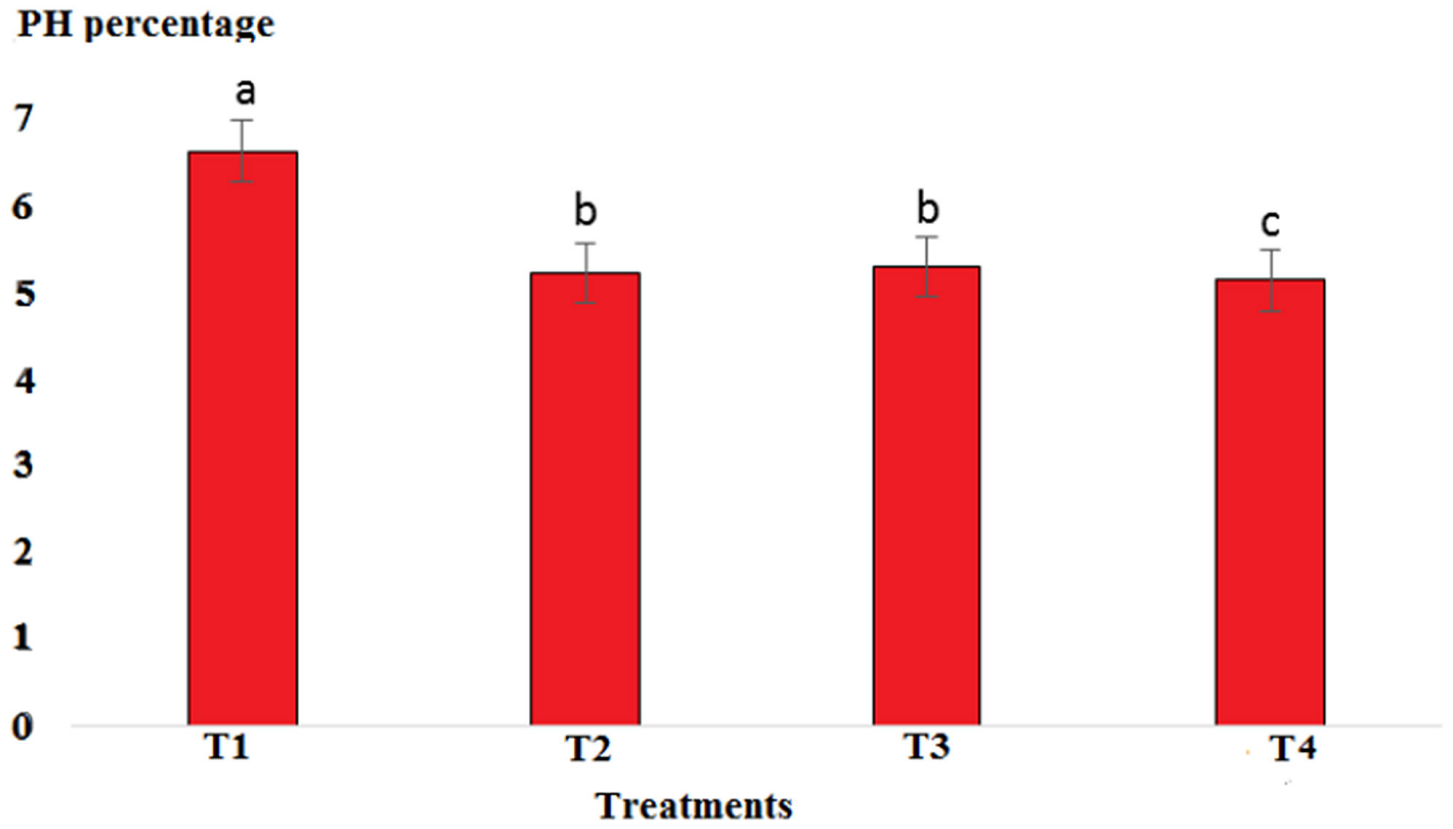
Comparison of pH values of different cooked marinade treatments with control. T1 was the control. For three treatments, T2, T3, and T4, 25 g of lemon juice, 12.5 g of freshly grated garlic, 3 g of salt, 0.15 g of turmeric, 0.05 g of red pepper powder, 0.15 g of black pepper powder, and 5 cc of distilled water were mixed and homogenized. The ratio of lemon juice and salt was 1 to 2. For treatment 1, lemon juice 4% and salt 8%; for treatment 2, lemon juice 5% and salt 10%; and for treatment 3, lemon juice 6% and salt 12% were used.

**FIGURE 8:**
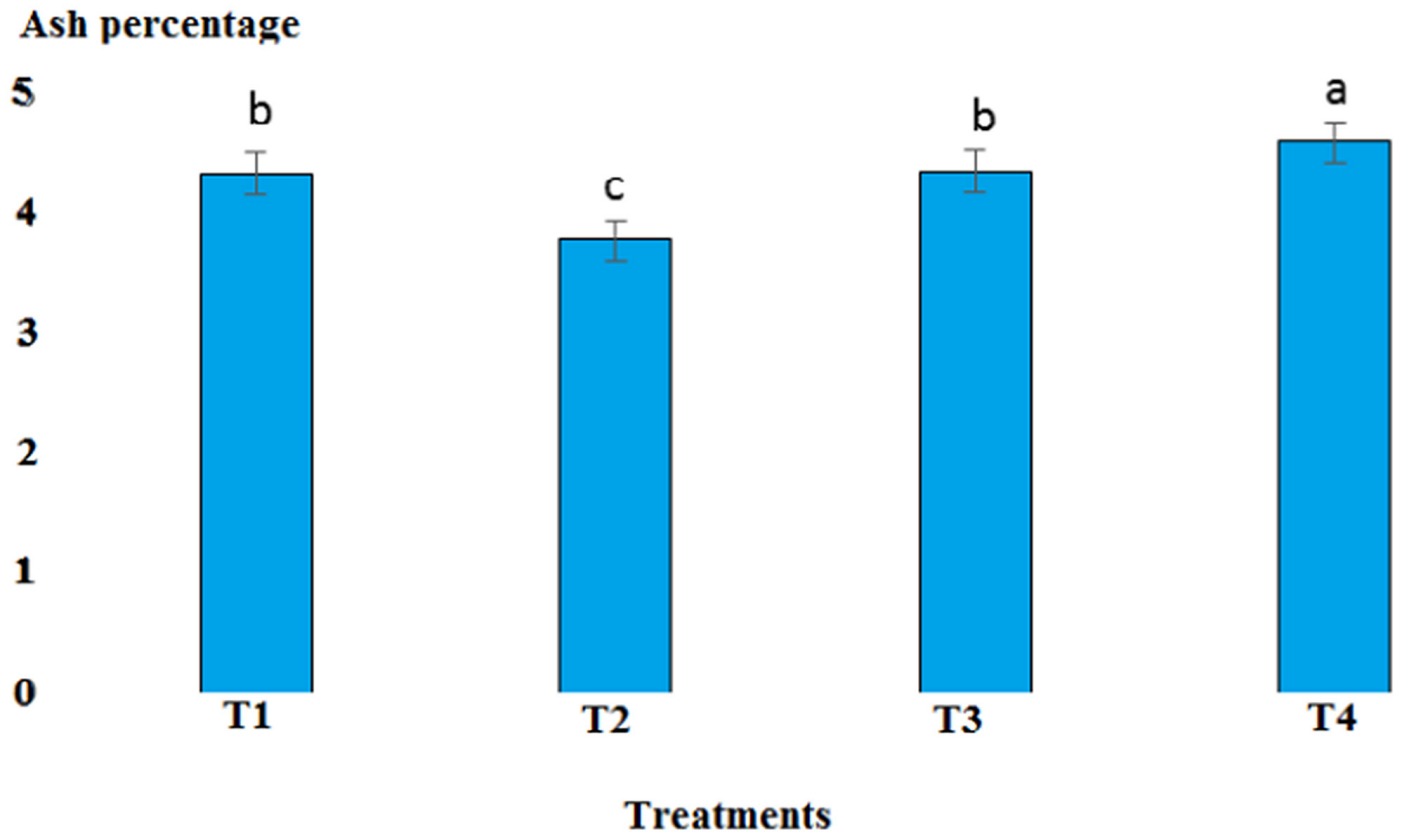
Comparison of ash contents of different cooked marinade treatments with control. T1 was the control. For three treatments, T2, T3, and T4, 25 g of lemon juice, 12.5 g of freshly grated garlic, 3 g of salt, 0.15 g of turmeric, 0.05 g of red pepper powder, 0.15 g of black pepper powder, and 5 cc of distilled water were mixed and homogenized. The ratio of lemon juice and salt was 1 to 2. For treatment 1, lemon juice 4% and salt 8%; for treatment 2, lemon juice 5% and salt 10%; and for treatment 3, lemon juice 6% and salt 12% were used.

**FIGURE 9:**
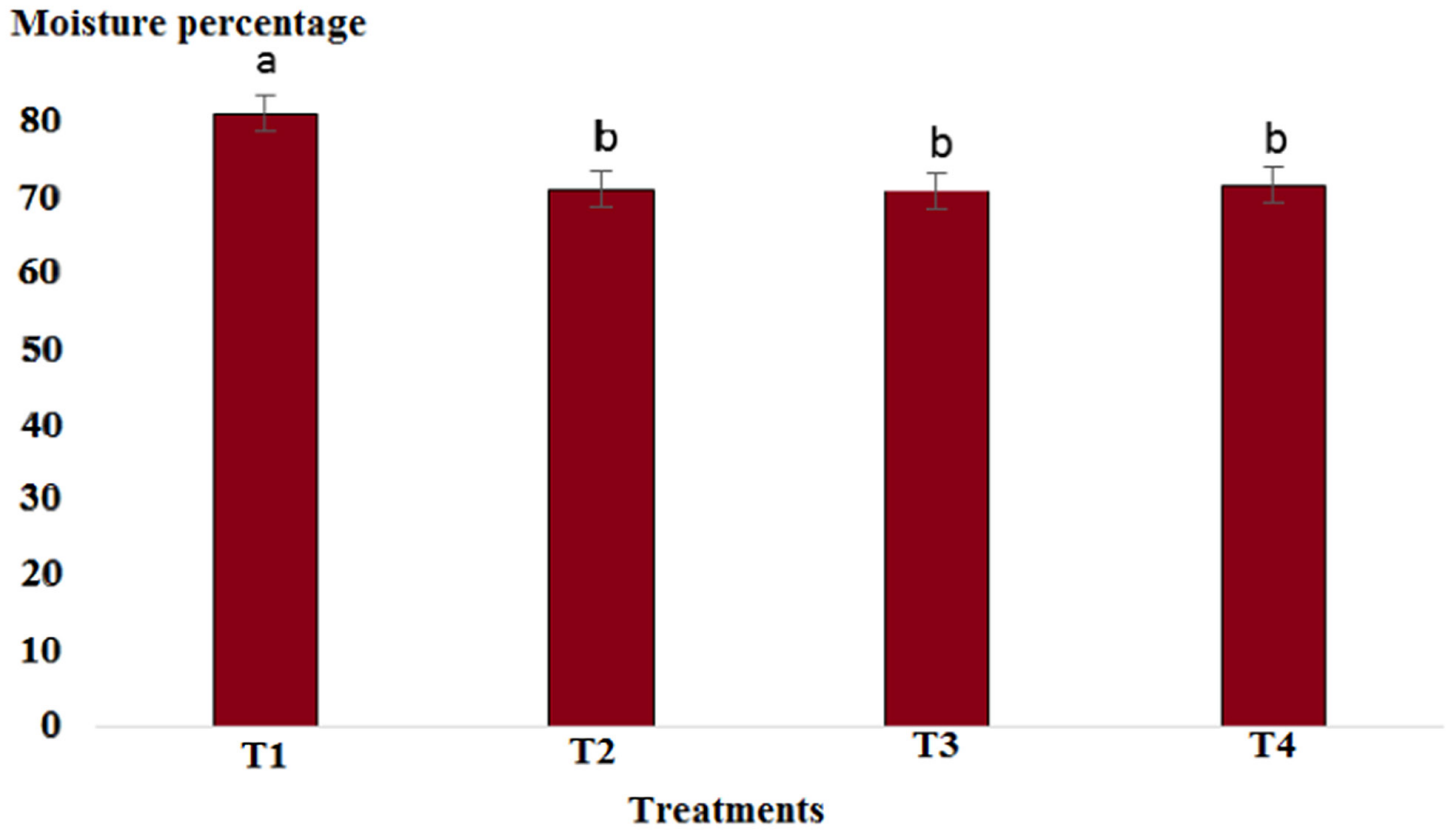
Comparison of moisture contents of different cooked marinade treatments with control. T1 was the control. For three treatments, T2, T3, and T4, 25 g of lemon juice, 12.5 g of freshly grated garlic, 3 g of salt, 0.15 g of turmeric, 0.05 g of red pepper powder, 0.15 g of black pepper powder, and 5 cc of distilled water were mixed and homogenized. The ratio of lemon juice and salt was 1 to 2. For treatment 1, lemon juice 4% and salt 8%; for treatment 2, lemon juice 5% and salt 10%; and for treatment 3, lemon juice 6% and salt 12% were used.

**FIGURE 10:**
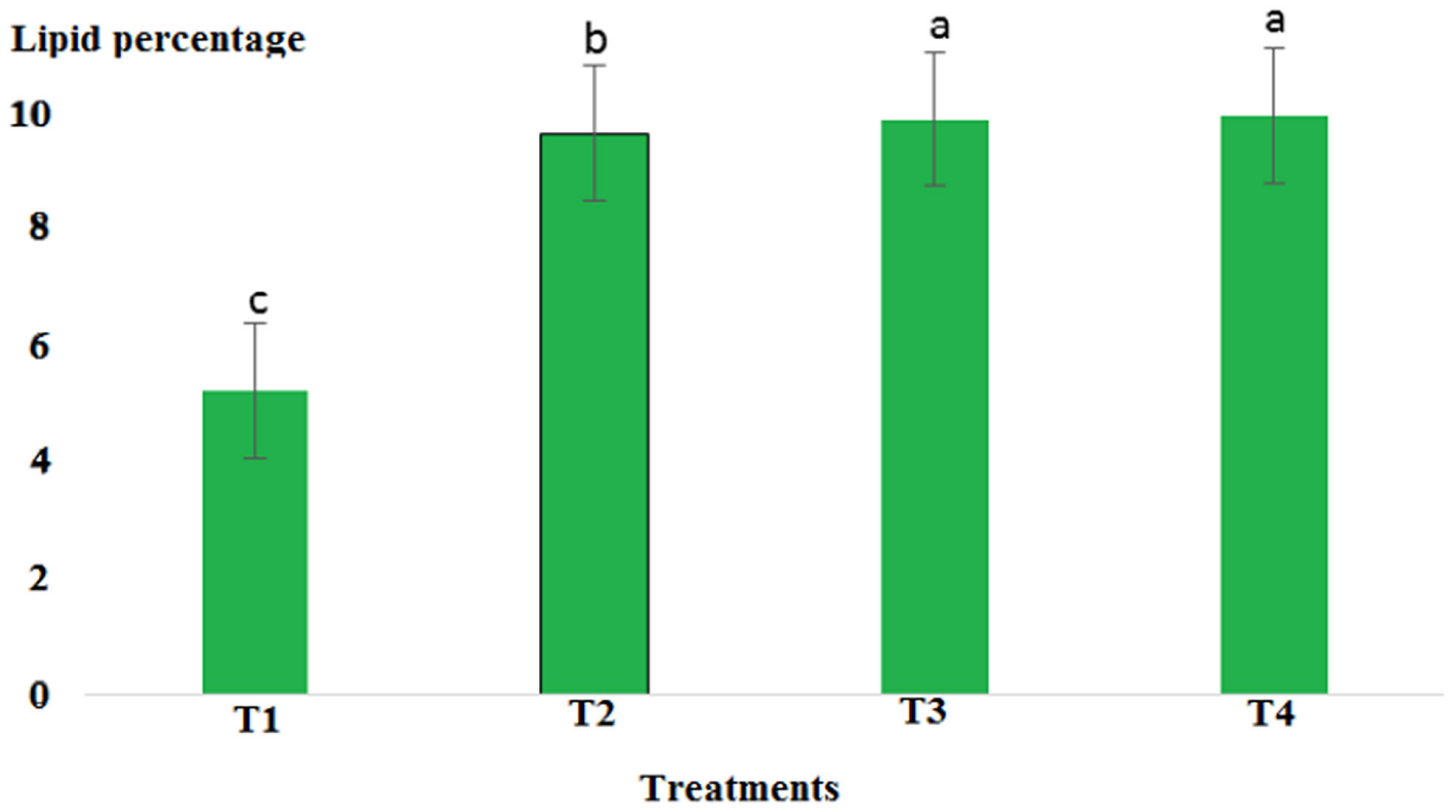
Comparison of lipid contents of different cooked marinade treatments with control. T1 was the control. For three treatments, T2, T3, and T4, 25 g of lemon juice, 12.5 g of freshly grated garlic, 3 g of salt, 0.15 g of turmeric, 0.05 g of red pepper powder, 0.15 g of black pepper powder, and 5 cc of distilled water were mixed and homogenized. The ratio of lemon juice and salt was 1 to 2. For treatment 1, lemon juice 4% and salt 8%; for treatment 2, lemon juice 5% and salt 10%; and for treatment 3, lemon juice 6% and salt 12% were used.

The pH value in the cooked marinade for control with 6.65 was highest, while in T2, it was 5.24, in T3, it was 5.32, and in T4, it was 5.17. There was a significant difference between T4 and the other treatments (*p* < .05). A sharp decrease in the pH level in cooked marinade can be due to the effect of cooking on the inactivation of microorganisms and enzymes (Figure 7). The ash content in cooked marinade for T4 with 4.6% was highest, while in T2, it was 3.78%, and in T3, it was 4.38%. There was a significant difference between T2 and T4 compared to the control (*p* < .05). The increase in the ash content was due to the absorption of flavoring and coloring compounds in the marinade solution in the fillet (Figure 8). The moisture content in cooked marinade for T4 with 71.66% was highest, while in T2, it was 71%, and in T3, it was 70.8% without significant difference (*p* < .05). There was a slight difference in the moisture content in the treatments, which was due to the balance of the moisture‐absorbing material and the infiltration of water in the fillet (Figure 9). The lipid content in cooked marinade for T4 with 9.98% was highest, while in T2, it was 9.68%, and in T3, it was 9.9%. There was a significant difference between T2 and T4 compared with the control (*p* < .05). The increase in fat content was due to an inverse ratio between moisture and fat contents (Figure 10). The highest calorie in the cooked marinade was related to T1 without being significantly different from other treatments (*p* < .05). The lowest weight loss in cooked marinade was related to treatment 2 (Table 2). The highest calorie in cold marinade was related to treatment 3, and the highest weight increase in cold marinade was related to treatment 2 (Table 3).

**TABLE 2:** Energetic values, moisture retention, and marinade yield of control and treatments in cooked marinade.

Samples	Control	Treatment 1	Treatment 2	Treatment 3
Energetic values (kcal)	63.60	157.04	156.47	152.29
Energetic values (KJ)	267.11	659.57	657.17	639.62
Marinade yield (weight decrease percentage)	—	19.28 ± 0.11	7.89 ± 0.08	13.96 ± 0.21
Moisture retention %	—	13.5	5.52	9.77

**TABLE 3:** Energetic values and marinade yield of control and treatments in cold marinade.

Samples	Control	Treatment 1	Treatment 2	Treatment 3
Energetic values (kcal)	81.52	71.21	62.13	83.59
Energetic values (KJ)	342.34	299.08	260.95	351.08
Marinade yield (weight increase percentage)	—	27.1 ± 0.18	27.6 ± 0.12	21.61 ± 0.23

The additive and synergistic effects of the marinating composition relate to preserving the Rainbow trout fillets and thus increasing the shelf life of the product. The main ingredients used in marinade are vinegar, salt, and spices such as garlic and pepper. Using vinegar as a food preservative is a traditional way to prevent spoilage because it is an effective acidulant and decreases the pH below the growth range of many bacteria (Jay, 2000). Salt is added to foods because of its effects on sensory, functional, and storage properties.

Different pH levels can be useful to evaluate the quality changes of fish during storage. The initial pH of fresh fish meat in the current study was in the neutral range of 6.5. The decrease in pH in stored fish meat is due to acidification, which is a common metabolite of the growth of a number of bacteria, including lactic acid bacteria. These results are consistent with the results of Maktabi et al. (2015), who used the same marinating process method as the present study.

The initial pH values of sardine 5.83 to 6.2, anchovy 5.8, and rainbow salmon 6.7 have been reported. In the present study, the initial pH value of the pickled samples (5.6) was significantly (*p* < .05) lower than that of the control samples (6.4) and was almost stable during storage. This difference can be due to the use of acetic acid (lemon juice) for pickling fish. A similar result to our findings was observed by Ozogul et al. (2010), in which the factor was stable in marinated anchovies after 6 months of storage.

A good example of barrier technology is fermentation. It acts as a protective strategy by lowering the pH and redox potential (Eh) of the substrate. Fermentation is sometimes called biopreservation in modern cooking methods. These active compounds prevent the growth of pathogenic and spoilage microorganisms and help protect the fish.

In recent years, the use of modern fermentation processes for fish has attracted the attention of an increasing number of researchers, which has changed the organoleptic properties of the products. Therefore, it is important to demonstrate the mechanism of biogenic amine formation and control during the fermentation of fish marinade. A new method to control biogenic amines in fermented foods was discovered through the participation of microorganisms that control biogenic amines (Jones et al., 2021). Results in present study showed that the pH level in treatments 3 and 4 was lower due to decreased amine compounds. Reducing the pH in treatments means reducing the production of nitrogenous compounds in the product, which indicates a reduction of enzymatic and microbial spoilage in the marinade.

Results of one study showed that proximate composition and calorific values decreased in the marinated *O. mykiss* fish, except for ash and fat. This was due to the effects of acid, salt, and other additives on the fish because they are moisture absorbers. As moisture content decreased, other nutrients increased. The decrease in moisture content can be due to the presence of salt in the marinade sample, which may have replaced part of the water in the raw material (Aberoumand. & Baesi., 2022). Protein in the marinade was significantly lower (*p* < .05) and was lowest in T3 and T4 samples. The moisture content decreased, which was due to the loss of water and the penetration of salt into the fillet, which led to an increase in the protein content (Aberoumand. & Baesi., 2022). Marinades have a significantly higher amount of crude protein (*p* < .05) because these treatments contain vinegar, which may help the process of “carination” faster, thus increasing the production of protein compounds in fish fillets. The protein values of the samples in this study were determined at 14.50% and 15.50% for control and marinade, respectively. This is due to the removal of moisture and water‐soluble proteins by the salt from the fillet during processing.

The chemical composition of the fish muscle is related to sex, age, environment, and season (Huss, 1995). It is important to determine the chemical composition of fish for processors to know the nature of the raw material before adequate processing techniques can be correctly applied. The proximate composition, on a dry matter basis, of the control and marinades is shown in Figures 2, 3, 4, 5, 6 and 8, 9, 10. Significant differences were detected in the moisture and protein contents of the control and marinated samples (*p* < .05), while no significant differences were observed for lipid contents (*p* < .05). It can be observed that the moisture content significantly decreased (*p* < .05) when the raw fillets were processed in different marinades. The decrease in moisture content might be attributed to the presence of organic salts in the marinades, which may have replaced some of the water present in the raw material (Adepoju et al., 2018; Espejo‐Hermes, 1998). Protein was found to be significantly higher (*p* < .05) in T1. Processing methods such as marinating can cause fish fillets to lose water with a consequent increase in protein content (Colakoglu et al., 2011), which was in agreement with the present study results in cooked marinade treatments 2 and 3. This may be due to the considerable proteolysis that may have occurred as a result of enzymatic activity during the “ripening” process, and high proteinaceous compounds were produced in the meat (Espejo‐Hermes, 1998; Tokur, 2007). Marinade samples have significantly high crude protein contents (*p* < .05) because these treatments contained vinegar, which may have contributed to a faster “ripening” process, thus increasing the production of proteinaceous compounds in fish fillet. The results obtained in the present study were in agreement with those of Sallam et al. (2007), who observed that the marinating process in Pacific saury (*Cololabis saira*) fillets decreased the moisture content and increased the other components analyzed compared with the control raw samples.

Szymczak et al. (2018) reported that in the first 100 h of the marinating process, the fastest increase in the content of peptides is observed, which is due to the activity of endopeptidases, while after 5–7 days, the increase in the content of free amino acids may increase as a result of the activity of exopeptidases, which in the present study during the marinating process for 4 days. Sample analysis was performed on the 1st and 4th days of storage, which agreed with the results of Szymczak et al. (2018) because the fastest increase in the content of peptides and bioactive compounds was due to the activity of endopeptidases.

The results of Kindossi et al. (2016) showed that the salt ratio, citric acid concentration, and marination time affect the microbiological and chemical quality of marinated fish flesh significantly (*p* ≤ .05). The optimum marinating conditions based on desirable pH values in the sample were established as a salt ratio of 10 g/100 g, a citric acid concentration of 2.5 g/100 g, and a marination time of 6 h. These conditions present the best quality of marinated fish flesh, leading to the safety of the final fermented product, which agreed with treatment 3 in which lemon juice 5% and salt 10% were used in the present study.

Rostamani et al. (2021) reported that increased marinating times of 24 h were found to produce more tender beef meat with increased WHC, soluble collagen, moisture contents, and decreased required force for shearing marinated beef meat cuts. These results have highlighted the efficiency of a short marinating time of 24 h to accomplish the reduced level of required shear force and high level of moisture maintenance, which resulted in high tenderness and customer acceptance. To conclude, a strong positive correlation with the drip loss (*p* < .01) and a significant negative correlation with the moisture content (*p* < .01) affected the tenderness of marinated beef meat, which approximately agreed with the cooked marinade results in the present study.

The cooking loss values of meat marinated with vinegar were significantly lower than those of the control group. It increased the texture scores. It could be concluded that organic acid containing these fruit vinegars can be a natural marinating liquid in old cow meat marinating for improving consumer acceptability (Unal et al., 2023), which was agreed with the cold marinade results in this study.

### 3.2 Mechanism of marinades during marinating

Using marinade ingredients as an effective preservative in marinating is the oldest and most common method in the meat industry. Marinades enhance flavor, retain moisture, improve texture, inhibit bacterial growth, and improve yield. They are constantly used in the fish meat sector because they dissolve easily in water and the ionic strength of water increases rapidly. Different types of fish meat have approximately 80% moisture, while muscle tissue fluid has a lower ionic strength than the marinade solution. The marinade solution is easily absorbed by the fish products by osmotic processes until equilibrium is reached. Functional components of additives are used in marinades to achieve different functions in final products. The main properties achieved by the functional ingredients of marinades are their binding properties (improved adhesion properties between meats and meat pieces), texture modification (improved tenderness), fat, and WHC. In addition, they reduce formulation costs by adding water to meat and meat products, increasing processing efficiency, or allowing the use of inexpensive raw meat sources in various product formulations. Myofibrillar proteins are primarily responsible for the textural properties and WHC of meat products. They can determine how marinades can improve the characteristics of the meat. In addition, pickling, which involves soaking the marinade solution in the meat, improves the flavor and aroma. First, the salt removes the liquid from the meat through osmosis. Then, the marinade solution is absorbed into the meat as the muscle structure breaks down. This solution pulls water‐soluble flavors such as garlic and onion below the surface of the meat. Oils also help transfer fat‐soluble flavors from seasonings such as pepper, herbs, and some spices to the surface of the fish meat (Ehsanur Rahman et al., 2023). In the present study, the moisture content of the studied fish was 78.5%, which reached 77.5% due to the marinating process in treatment 4 due to the effects of lemon juice, freshly grated garlic, salt, turmeric, red pepper powder, and black pepper powder. The decrease in protein percentage in the finished product was due to the effect of protease enzymes on fish protein. The increase in ash percentage in treatment 4 was due to the use of additives for the process, and the increase in fat percentage in treatment 4 was caused by the use of salt and acid additives that reduced moisture content.

A slight decrease in pH level was observed at the end of the storage period for both control and marinated samples; however, such a decrease in pH level was higher in the control samples than in the marinated samples. This finding is not in agreement with those reported for marinated anchovies, marinated sardines, and marinated Saury (Sallam et al., 2007) during storage at refrigerated temperature. During the storage of marinades, heterofermentative lactic acid bacteria can grow and degrade the amino acids with the formation of carbon dioxide and other decarboxylation products, which bind acetic acid and raise the pH of marinades (Simora et al., 2021).

In cooked marinade, the product yield was low due to weight loss, while in cold marinade, the yield was high due to weight increase. In cold marinade, the highest weight increase is related to treatment 2, but in cooked marinade, the lowest weight loss is related to treatment 2. The maximum calorie of cold marinades is related to treatment 3, and the maximum calorie of cooked marinades is related to treatment 1. Therefore, it can be concluded that treatment 2 was the best option in both cold and cooked marinade preparation methods because the compounds in the marinade solution in treatment 2 can better retain macronutrients and the product yield is as high as possible. A literature review showed that proximate analysis were not significant differences in the whole‐body fish *O. mykiss* composition for moisture, fat, or energy (dry and wet) was affected experimental diet compared with the control diet.

Moisture retention of the fish‐cooked marinade is given in Table 2. Moisture retention of fish‐cooked marinade samples was determined at 13.5%, 5.52%, and 9.77%. The moisture retention of the fish marinade by adding additives was decreased (*p* < .05). These differences could be due to the differences in moisture content for all samples of cooked marinade. The highest moisture retention was found in treatment 1 (13.5%).

## 4 CONCLUSIONS

Results showed protein content in cooked marinade treatment 2 (15.54%) was highest, while in treatments 3 and 4, it was 14.92% and 13.76%, respectively, with a significant difference (*p* < .05). The lipid content in cooked marinade T4 (9.98%) was highest, while in treatments 2 and 3, it was 9.68% and 9.9%, respectively, with a significant difference (*p* < .05). The highest calorie value in cooked marinade was related to treatment 1, without being significantly different (*p* < .05). The highest moisture retention in cooked marinades was found in treatment 1 (13.5%).The lowest weight loss in cooked marinade was related to treatment 2. The highest calorie value in cold marinade was related to treatment 3, and the highest weight increase in cold marinade was related to treatment 2. It can be concluded that treatment 2 was the best option in both cold and cooked marinade samples; due to the compounds that exist in the marinade solution in treatment 2, which can better preserve nutrients, the yield was as high as possible. T2 found the highest protein and the lowest fat, as well as the highest moisture percentage and pH value. In this method, flavored ready‐to‐eat fish fillet could be available for consumers.

## AUTHOR CONTRIBUTIONS

**Ali Aberoumand:** Conceptualization (equal); data curation (equal); formal analysis (equal); funding acquisition (equal); investigation (equal); methodology (equal); project administration (equal); resources (equal); software (equal); supervision (equal); validation (equal); visualization (equal); writing – original draft (equal); writing – review and editing (equal). **Abolfazl Aminimehr:** Formal analysis (equal); investigation (equal).

## FUNDING INFORMATION

This work was supported by Behbahan Khatam Alanbia University of Technology, Behbahan, Iran.

## CONFLICT OF INTEREST STATEMENT

The authors do not have any conflicts of interest.

## ETHICAL APPROVAL

This study did not involve human or animal testing.

## INFORMED CONSENT

Written informed consent was obtained from all study participants.

## Data Availability

The datasets used are available from the corresponding authors upon reasonable request.

## References

[fsn33745-bib-0001] Aberoumand, A. , & Baesi, F. (2022). Valuation of fatty acid‐related nutritional quality indices in processed and raw (*Lethrinus lentjan*) fish fillets. Food Science & Nutrition, 11(2), 936–971. 10.1002/fsn3.3131 PMC9922119 36789051

[fsn33745-bib-0002] Adepoju, M. A. , Omitoyin, B. O. , Ajani, E. K. , & Asha, K. (2018). Effect of smoking time and temperature on the proximate composition and quality of milkfish steaks. Journal of Aquatic Food Product Technology, 27, 369–378. 10.1080/10498850.2018.1437494

[fsn33745-bib-0003] AOAC . (2005). Association of Official Analytical Chemists Official Methods of analysis of the Association of Official Analytical Chemists, international (18th ed.). AOAC.

[fsn33745-bib-0004] Colakoglu, F. A. , Ormanci, H. S. , & Cakir, F. (2011). Effect of marinating and smoking on lipid and fatty acid composition of thornback ray (*Raja clavata*) and spiny dogfish (*Squalis acanthias*). European Food Research Technology, 232, 1069–1075. 10.1007/s00217-011-1477-x

[fsn33745-bib-0005] Ehsanur Rahman, S. M. , Islam, S. , Pan, J. , Kong, D. , Xi, Q. , Du, Q. , Yang, Y. , Wang, J. , Oh, D. H. , & Han, R. (2023). Marination ingredients on meat quality and safety—A review. Food Quality and Safety, 2023(7), 1–17. 10.1093/fqsafe/fyad027

[fsn33745-bib-0006] Espejo‐Hermes, J. (1998). Fish processing in the tropics (p. 336). Tawid Publication.

[fsn33745-bib-0007] Gamage, H. G. C. L. , Mutucumarana, R. K. , & Andrew, M. S. (2017). Effect of marinating method and holding time on the physicochemical and sensory characteristics of broiler meat. The Journal of Agricultural Sciences, 12(S3), A172–A184.

[fsn33745-bib-0008] Gomez, I. , Ibañez, F. C. , & Beriain, M. J. (2019). Physicochemical and sensory properties of sous vide meat and meat analog products marinated and cooked at different temperature‐time combinations. International Journal of Food Properties, 22(1), 1693–1708.

[fsn33745-bib-0009] Gomez, I. , Janardhanan, R. , & Ibanez, F. C. (2020). The effects of processing and preservation technologies on meat quality: Sensory and nutritional aspects. Food, 9(10), 1416. 10.3390/foods9101416 PMC7601710 33036478

[fsn33745-bib-0010] Gómez‐Salazar, J. A. , Ochoa‐Montes, D. A. , & Cerón‐García, A. (2018). Effect of acid marination assisted by power ultrasound on the quality of rabbit meat. Journal of Food Quality, 2018, 1–6.

[fsn33745-bib-0011] Halpern, B. S. , Frazier, M. , & Afflerbach, J. (2019). Recent pace of change in human impact on the world's ocean. Scientific Reports, 9, 11609. 31406130 10.1038/s41598-019-47201-9 PMC6691109

[fsn33745-bib-0012] Huss, H. H. (1995). Quality and quality changes in fresh fish (p. 348). FAO.

[fsn33745-bib-0013] Jay, J. M. (2000). Modern food microbiology (6th ed., pp. 120–131). Aspen Publishers.

[fsn33745-bib-0014] Jones, N. A. R. , Webster, M. , & Salvanes, A. G. V. (2021). Physical enrichment research for captive fish: Time to focus on the DETAILS. Journal of Fish Biology, 99, 704–725. 33942889 10.1111/jfb.14773

[fsn33745-bib-0015] Kindossi, J. M. , Anihouvi, V. B. , Vieira‐Dalodé, G. , Akissoé, N. H. , & Hounhouigan, D. J. (2016). Optimization of the marinating conditions of cassava fish (*Pseudotolithus* sp.) fillet for Lanhouin production through application of Doehlert experimental design. Food Science & Nutrition, 4(2), 261–268. 10.1002/fsn3.285 27004115 PMC4779494

[fsn33745-bib-0016] Kusi, P. (2023). Analysis of the production and quality of marinated fish with potential perspectives. Journal of Fisheries Sciences, 17(2), 126–129.

[fsn33745-bib-0017] Larsen, R. , Olsen, S. H. , Katsuwonus, S. , & Elvevoll, E. O. (2008). Low salt brining of pre‐rigour filleted farmed cod (*Gadus morhua* L.) and the effects on different quality parameters. LWT‐Food Science and Technology, 41(7), 1167–1172. 10.1016/J.LWT.2007.07.015

[fsn33745-bib-0018] Latoch, A. (2020). Effect of meat marinating in kefir, yoghurt and buttermilk on the texture and color of pork steaks cooked sousvide. Annals of Agricultural Sciences, 65(2), 129–136.

[fsn33745-bib-0019] Latoch, A. , Libera, J. , & Stasiak, D. M. (2019). Physicochemical properties of pork loin marinated in kefir, yoghurt or buttermilk and cooked sous vide. Acta Scientiarum Polonorum Technologia Alimentaria, 18(2), 163–171. 31256544 10.17306/J.AFS.0642

[fsn33745-bib-0020] Lopes, S. M. , da Silva, D. C. , & Tondo, E. C. (2022). Bactericidal effect of marinades on meats against different pathogens: A review. Critical Reviews in Food Science and Nutrition, 62(27), 7650–7658. 33905272 10.1080/10408398.2021.1916734

[fsn33745-bib-0021] Maktabi, S. , Zarei, M. , & Chadorbaf, M. (2015). Effect of traditional marinating on bacterial and chemical characteristics in frozen rainbow trout fillet. Journal of Food Quality and Hazards Control, 2, 128–133.

[fsn33745-bib-0022] Maktabi, S. , Zarei, M. , & Chadorbaf, M. (2016). Effect of a traditional marinating on properties of rainbow trout fillet during chilled storage. Veterinary Research Forum, 7(4), 295–300. 28144420 PMC5251351

[fsn33745-bib-0023] Österblom, H. , Crona, B. I. , Folke, C. , Nyström, M. , & Troell, M. (2017). Marine ecosystem science on an intertwined planet. Ecosystems, 20, 54–61.

[fsn33745-bib-0024] Ozogul, Y. , Ozogul, F. , & Kuley, E. (2010). Effects of combining of smoking and marinating on the shelf life of anchovey stored at 4°C. Food Science and Biotechnology, 19, 69–75.

[fsn33745-bib-0025] Ozturk, B. , & Sengun, I. Y. (2019). Inactivation effect of marination liquids prepared with koruk juice and dried koruk pomace on *salmonella Typhimurium*, *Escherichia coli* O157:H7 and listeria monocytogenes inoculated on meat. International Journal of Food Microbiology, 304, 32–38. 31152975 10.1016/j.ijfoodmicro.2019.05.013

[fsn33745-bib-0026] Rostamani, M. , Baghaei, H. , & Boland, M. (2021). Prediction of top round beef meat tenderness as a function of marinating time based on commonly evaluated parameters and regression equations. Food Science & Nutrition, 9(9), 5006–5015. 10.1002/fsn3.2454 34532012 PMC8441426

[fsn33745-bib-0027] Sallam, K. , Ahmed, A. M. , Elgazzar, M. M. , & Eldaly, E. A. (2007). Chemical quality and sensory attributes of marinated Pacific saury (*Cololabis saira*) during vacuum‐packaged storage at 4°C. Food Chemistry, 102(4), 1061–1070. 10.1016/j.foodchem.2006.06.044

[fsn33745-bib-0028] Simora, R. M. C. , Armada, C. D. , & Babaran, R. P. (2021). Quality assessment of marinated flying fish (*Cheilopogon intermedius*) fillets during vacuum‐packed storage at 4°C. Philippine Journal of Science, 150(1), 223–232.

[fsn33745-bib-0029] Sushri, S. , Madathil, B. D. , Verma, S. K. , & Pathak., N. (2020). Seafood marinating‐a review. International Archive of Applied Sciences and Technology, 11(3), 165–168. 10.15515/iaast.0976-4828.11.3.165168

[fsn33745-bib-0030] Szymczak, M. , Felisiak, K. , & Szymczak, B. (2018). Characteristics of herring marinated in reused brines after microfiltration. Journal of Food Science and Technology, 55(11), 4395–4405. 10.1007/s13197-018-3343-3 30333635 PMC6170335

[fsn33745-bib-0031] Tokur, B. (2007). The effect of different cooking methods on proximate composition and lipid quality of rainbow trout (*Oncorhynchus mykiss*). International Journal of Food Science and Technology, 42, 874–879. 10.1111/j.1365-2621.2006.01298.x

[fsn33745-bib-0032] Unal, K. , Babaoğlu, A. S. , & Karakaya., M. (2023). Improving the textural and microstructural quality of cow meat by black chokeberry, grape, and hawthorn vinegar‐based marination. Food Science & Nutrition, 1–11. 10.1002/fsn3.3566 PMC10563726 37823113

